# Risk prediction models for extubation failure in critically ill patients on mechanical ventilation: a systematic review

**DOI:** 10.3389/fmed.2025.1695394

**Published:** 2025-11-20

**Authors:** Xiang Zeng, Xiao Juan Chen, Ping Lai, Jie Chen, Zhoujing Chen, Xiyu Qi

**Affiliations:** Chongqing JiangJin District Hospital of Chinese Medicine, Chongqing, China

**Keywords:** Intensive Care Unit, mechanical ventilation, extubation failure, risk prediction model, systematic review

## Abstract

**Background:**

Failure to extubate successfully from mechanical ventilation is a critical event associated with poor prognosis in ICU patients, significantly prolonging hospital stays and increasing mortality rates. It is widely accepted in academic circles that developing prediction models for extubation failure can facilitate precise extubation decisions. Despite the rapid proliferation of relevant prediction models, their methodological quality and bedside applicability remain ambiguous.

**Objective:**

This study aims to outline the predictive factors associated with the risk of extubation failure in patients undergoing mechanical ventilation in the Intensive Care Unit (ICU) and to summarize the existing predictive models.

**Methods:**

We searched the China National Knowledge Infrastructure (CNKI), Wanfang Database, VIP Database, China Biomedical Database, PubMed, Embase, Web of Science, and Cochrane Library. We included both prospective and retrospective studies that developed or validated risk prediction models for extubation failure in patients undergoing mechanical ventilation in the ICU. The Prediction Model Risk of Bias Assessment Tool (PROBAST) was used to assess the bias and applicability of the models.

**Results:**

This analysis includes 14 studies. Frequency analysis of the predictors revealed that there are 15 predictors that appeared at least twice, among which mechanical ventilation duration, GCS score, APACHE II score, age, and hemoglobin were the most common predictors. From the perspective of the models, only 2 studies conducted both internal and external validation, 3 studies ultimately employed machine learning, while 11 studies utilized traditional modeling methods. However, we found that many studies faced issues such as insufficient sample sizes, missing crucial methodological information, and all models being rated as having a high risk of bias.

**Conclusion:**

Most published predictive models lack methodological rigor, leading to a heightened risk of bias. Future research should prioritize the enhancement of methodological rigor and the external validation of risk prediction models for extubation failure in ICU patients receiving mechanical ventilation. Additionally, it is essential to emphasize adherence to scientific methods and transparent reporting to improve the accuracy and generalizability of research findings.

**Systematic review registration:**

https://www.crd.york.ac.uk/PROSPERO/recorddashboard, Registration number:CRD420251124371.

## 1 Introduction

The Intensive Care Unit (ICU) is a department that focuses on the centralized treatment of critically ill patients (1). Due to the critical condition of severely ill patients, their ability to maintain spontaneous breathing is significantly diminished. When patients exhibit respiratory insufficiency, there is a risk of hypoxia, or they may have already shown signs of hypoxia; thus, mechanical ventilation treatment becomes necessary (2). Mechanical ventilation (MV) is one of the standard life support technologies in the ICU, with approximately 50% of ICU patients requiring MV (3). However, prolonged mechanical ventilation can lead to complications in patients, including Ventilator Associated Pneumonia (VAP), (4) barotrauma (5), airway injuries (6), and catheter-associated pressure injuries (7). During the treatment period, as the patient’s condition improves and respiratory function gradually returns to normal, the demand for mechanical ventilation support stabilizes and begins to decrease. Considering discontinuing mechanical ventilation and proceeding with extubation as early as possible is necessary.

Extubation, the gradual withdrawal of mechanical ventilation support, is a critical process through which critically ill patients regain their ability to breathe spontaneously and are liberated from the ventilator (8). This phase is essential for patients transitioning out of the intensive care unit. Extubation failure is the patient’s inability to sustain spontaneous breathing following extubation from the ventilator. This condition necessitates reconnection to the ventilator or occurs when spontaneous breathing lasts less than 48 h without ventilator support, requiring interventions such as non-invasive ventilation, high-flow oxygen therapy, re-intubation, terminal extubation, or tracheostomy (9). The offline process consists of three steps: offline screening, procedures, and extubation (10). The expert group of the Critical Care Medicine Branch of the Chinese Medical Association emphasizes in the “Clinical Application Guidelines for Mechanical Ventilation” that when the causes of respiratory failure in ICU patients are effectively controlled or improved, we should conduct weaning therapy as early as possible to achieve optimal therapeutic effects and prognosis (11). Determining the optimal timing for withdrawing mechanical ventilation is crucial in treatment. An appropriate extubation moment prevents unnecessary medical resource consumption and helps alleviate the financial burden on patients’ families. Related research reports that 5–30% of ICU patients experience weaning failure (12). Inappropriately delaying weaning from mechanical ventilation may increase the risk of complications such as pneumonia or ventilator-associated lung injury in patients on mechanical ventilation (13). This risk leads to increased medical costs and prolonged hospital stays for patients and may significantly elevate the overall mortality risk (14). Although successful extubation is an important goal in ICU treatment, an overly aggressive weaning process may lead to inadequate oxygen supply, respiratory muscle fatigue, and incomplete recovery of airway protective functions, which may increase the risk of extubation failure (15). It is noteworthy that extubation failure is not the result of a single pathological process, but rather the consequence of the combined effects of abnormalities across multiple systems, including the respiratory system (e.g., respiratory muscle fatigue, airway secretion retention) (16), the cardiovascular system (e.g., left ventricular overload) (17), neuromuscular function (e.g., myasthenia) (18), and metabolic status (e.g., malnutrition, frailty) (19).

Therefore, the early identification of high-risk populations for mechanical ventilation weaning failure in the ICU, along with timely and effective interventions for their risk factors, is of significant importance in reducing the incidence of mechanical ventilation extubation failure among ICU patients and improving clinical outcomes. Risk prediction models use mathematical formulas to assess the existence of specific conditions or the future risk of certain events, effectively identifying risk factors for diseases and quantifying the magnitude of risk associated with each factor (20). Multiple countries have developed various risk prediction models for extubation failure in ICU patients undergoing mechanical ventilation. However, these different models’ predictive capabilities and clinical applicability remain unclear. Furthermore, no studies have been found that systematically evaluate these models. Therefore, this study aims to systematically evaluate the risk prediction models for extubation failure in patients undergoing mechanical ventilation in the ICU, with the intention of providing a basis for clinical medical staff to select or develop appropriate risk prediction models for extubation failure in ICU mechanical ventilation patients.

## 2 Materials and methods

This systematic review has been registered in PROSPERO (Registration ID: CRD420251124371).

### 2.1 Inclusion and exclusion criteria

#### 2.1.1 Study types

Cohort studies, case–control studies, and cross-sectional studies.

#### 2.1.2 Research subjects

Patients aged ≥18 years requiring invasive mechanical ventilation in the ICU.

#### 2.1.3 Research content

The construction and/or validation of a prediction model for extubation failure in patients undergoing mechanical ventilation in the Intensive Care Unit.

#### 2.1.4 Exclusion criteria

① Non-Chinese or Non-English literature; ② Literature that only analyzes risk factors without establishing a risk prediction model; ③ Literature for which the original text cannot be obtained or data is incomplete; ④ Studies that have been published repeatedly; ⑤ Studies where the number of predictive variables included in the model is less than 2.

### 2.2 Literature retrieval strategy

A comprehensive search was conducted in various databases, including CNKI, Wanfang Data, China Biomedical Literature Database, VIP, PubMed, Web of Science, Embase, Cochrane Library, and CINAHL, regarding research on risk prediction models for extubation failure in patients on mechanical ventilation in the ICU. The search timeframe was from the establishment of the database until August 8, 2025. The search was restricted to English- and Chinese-language publications; no additional language filters were applied during the initial retrieval, but all non-English/non-Chinese articles were subsequently excluded in line with the pre-specified inclusion criteria. The search strategy combined both subject headings and free-text terms, focusing primarily on keywords such as “Intensive Care Units,” “ICU,” “Intubation, Intratracheal,” “Respiration, Artificial,” and “Risk Assessment.” This comprehensive approach ensures a thorough exploration of relevant literature in the context of critical care management. For the complete search strategy, please refer to Appendix A. Additionally, we employed the PICOTS framework recommended by the CHARMS checklist (21) for key evaluations and data extraction in systematic reviews to describe the key elements of this systematic review as follows. The detailed search strategy is provided in Appendix A.

P (Population, P): patients aged ≥18 years in the ICU receiving mechanical ventilation.

I (Intervention model, I): development and/or validation of a risk prediction model for extubation failure in ICU patients on mechanical ventilation.

C (Comparator, C): none. O (Outcome, O): The outcome is defined as extubation failure in patients on mechanical ventilation during their ICU stay.

T (Timing, T): before extubation in patients on mechanical ventilation in the ICU.

S (Setting, S): the intended use of this prediction model is for risk stratification in the ICU to assess the risk of extubation failure, thereby enabling timely preventive measures.

### 2.3 Literature screening and data extraction

Initially, two researchers (ZX and CXJ) independently screened the literature and extracted data based on inclusion and exclusion criteria. If necessary, a third reviewer (QXY) participated. The literature screening method involved using NoteExpress software to remove duplicate records, reading titles and abstracts for initial screening, excluding obviously irrelevant literature, and then further reading the full texts for secondary screening to determine the final included literature. Subsequently, standardized forms were developed for data extraction based on the Critical Appraisal and Data Extraction for Systematic Reviews of Prediction Modelling Studies (CHARMS) (21).

### 2.4 Assessment of bias risk in included studies

Two researchers employed the Prediction Model Risk of Bias Assessment Tool (PROBAST) (22) to evaluate the risk of bias and applicability of the models included in the literature.

#### 2.4.1 Bias risk assessment

PROBAST comprises four domains: study population, predictors, outcomes, and analysis. Each question can be answered as “Yes,” “Probably Yes,” “Probably No,” “No,” or “No Information.” If any domain is rated as “No” or “Probably No,” that domain is considered high risk; only when all questions are answered as “Yes” or “Probably Yes” is the domain considered low risk. If all four domains are assessed as low risk, the overall risk of bias (ROB) is rated as “Low”; if one or more domains are rated as uncertain risk while the remaining domains are low risk, the overall risk is classified as “Unclear.” The applicability assessment is similar to the bias risk assessment but uses only the first three domains to determine the applicability of the prediction model. The first two researchers (ZX and CXJ) conducted the assessments independently, with the final judgment made by a third reviewer (QXY).

#### 2.4.2 Applicability assessment

The applicability assessment encompasses three domains: the study subjects, the predictive factors, and the outcomes. The judgment process is similar to bias risk, where the overall applicability of the predictive model is rated as ‘low’, ‘high’, or ‘unclear’. The overall rating is deemed ‘low risk’ only when all domains are assessed as ‘low risk’. If one or more domains are rated as ‘high risk’, the applicability is classified as ‘high risk’. If a particular domain is rated as ‘unclear’, but all other domains are rated as ‘low risk’, the applicability is considered ‘unclear’.

## 3 Results

### 3.1 Literature screening process and results

**Figure 1:**
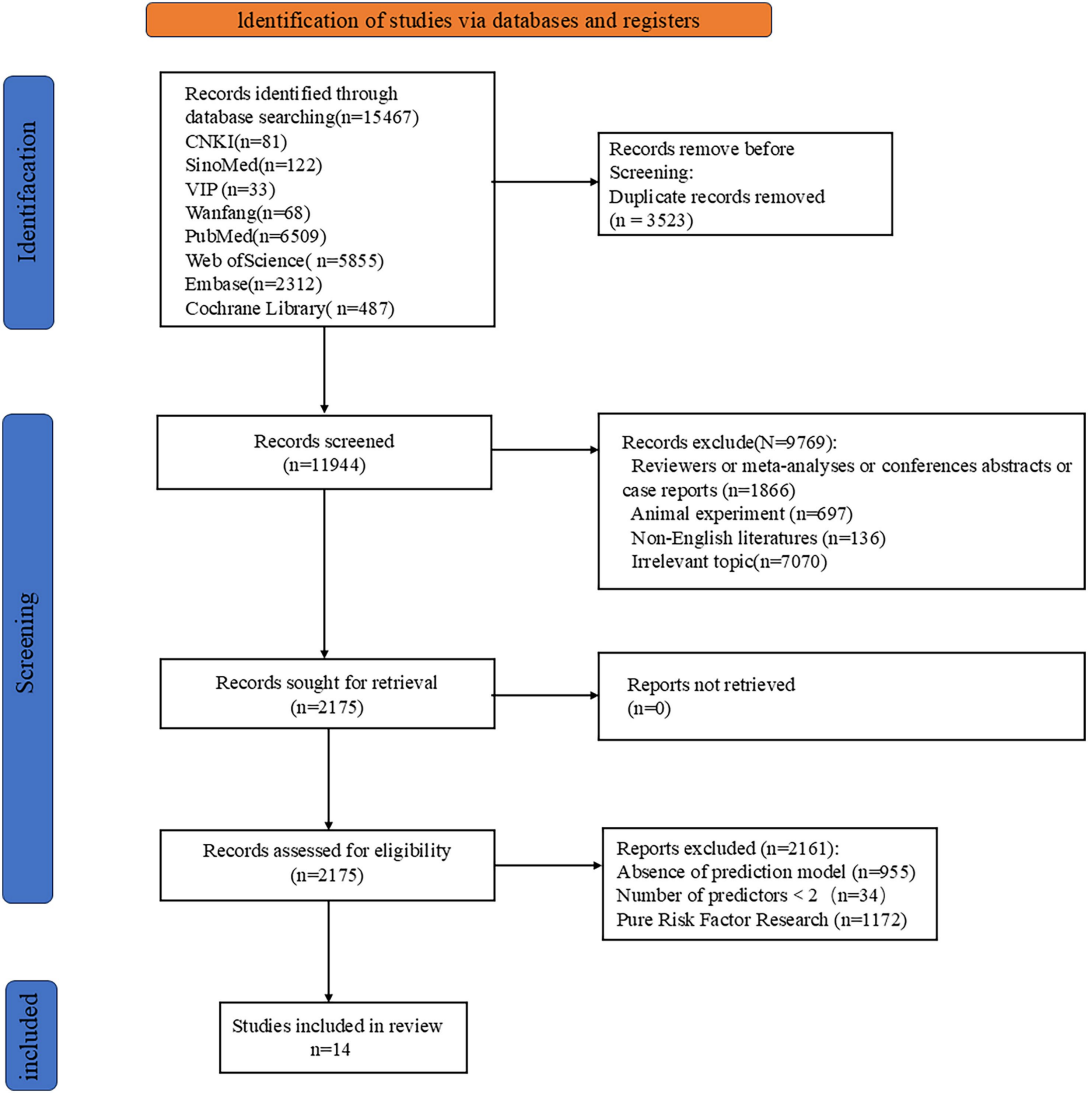
Flowchart of the literature search, screening, and final included.

A preliminary search yielded 15,467 relevant articles. After removing duplicates, 11,944 articles remained. A gradual screening process ultimately included 14 articles (23–36). The literature screening process and results are shown in Figure 1.

### 3.2 Basic characteristics of included studies and bias risk assessment results

Among the included literature are 8 studies from China (28, 30–36), 2 from the United States (26, 27), 1 from Brazil (25), 1 from Colombia (24), and 1 from France (23). Additionally, there is 1 multi-national collaborative study (29). In the past 5 years, 10 studies have been published (27–36). Among the 14 studies, 9 are retrospective studies (24, 27, 30–36), while 5 are prospective studies (23, 25, 26, 28, 29). The basic characteristics of the included literature are shown in Table 1.

**Table 1:** The basic characteristics of the included studies.

Author (year)	Country	Study design	Participants	Sample size		Outcome indicators
				Totality	Case	
Godet et al. (2017) (23)	France	Prospective study	MV patients with craniocerebral injury in the ICU	140	43	Re-intubation within 48 h after extubation
Sará-Ochoa et al. (2017) (24)	Colombia	Retrospective study	MV patients in the ICU	1,017	157	Re-intubation within 48 h after extubation
Dos Reis et al. (2017) (25)	Brazil	Prospective study	MV patients with traumatic brain injury in the ICU	311	43	Re-intubation within 48 h after extubation
Hsieh et al. (2018) (26)	USA	Prospective study	MV patients in the ICU	3,602	185	Re-intubation or death within 72 h after extubation
Bansal et al. 2022 (27)	USA	Retrospective study	MV patients in the ICU	6,161	746	Re-intubation within 72 h after extubation
Zhao et al. (2021) (28)	China	Prospective study	MV patients in the ICU	16,191	2,807	Re-intubation within 48 h after extubation
Cinotti et al. (2022) (29)	Multiple countries	Prospective study	MV patients in the ICU	1,512	231	Extubation failure within 5 days
Wang et al. (2023) (30)	China	Retrospective study	MV patients in the ICU	546	131	Need for non-invasive or invasive ventilatory support, or death, within 48 h after extubation
Li (2023) (31)	China	Retrospective study	MV patients in the ICU	548	230	Re-intubation within 48 h after extubation
Yang et al. (2023) (32)	China	Retrospective study	MV patients in the NICU	310	60	Re-intubation within 48 h after extubation
Zhao et al. (2023) (33)	China	Retrospective study	MV patients in the ICU	670	133	Death within 48 h after extubation or inability to resume spontaneous breathing within 48 h after extubation.
Hu et al. (2024) (34)	China	Retrospective study	MV elderly severe-pneumonia patients in the ICU	330	117	Requirement for non-invasive or invasive ventilatory support, or death, within 48 h after extubation.
Xu et al. (2024) (35)	China	Retrospective study	MV patients in the ICU	487	164	Re-intubation within 48 h after extubation
Sun et al. (2025) (36)	China	Retrospective study	MV patients in the EICU	138	11	Re-intubation within 48 h after extubation

### 3.3 Establishment of the models included

A total of 28 predictive models for the offline failure risk were reported in the studies included. The number of candidate predictive variables in each study ranged from 9 to 105. Regarding variable selection, 11 studies (23–27, 30–34, 36) employed univariate and multivariate analyses, 2 studies (28, 35) utilized recursive feature elimination, and 1 study (29) applied Lasso regression for variable selection. In the handling of continuous variables, 3 studies (32, 34, 36) converted continuous variables into categorical variables, while the remaining eleven studies (23–31, 33, 35) maintained the continuity of the continuous variables. In the area of missing data handling, 10 studies (25–27, 30–36) did not report the missing data and the methods used for handling it. 2 studies (28, 29) only reported the use of multiple imputation to supplement the missing data. However, it did not specify the exact number of missing data points. 1 study (23) directly deleted the missing data, while only 1 study (24) reported the missing data and the method of mean imputation employed. Regarding model establishment methods, 10 studies (23, 25, 27, 29–34, 36) utilized only Logistic Regression for modeling, while 1 study (26) employed Neural Networks for modeling. Another study (28) applied Machine Learning methods for modeling. Additionally, one study (35) utilized five methods: LR, RF, SVM, XG Boost, and Light GBM for modeling. 7 studies (23, 24, 26, 27, 29, 33, 35) conducted only internal validation, 2 studies (30, 36) performed only external validation, and 2 studies (28, 31) employed a combination of internal and external validation methods for evaluation. The remaining 3 studies (25, 32, 34) did not conduct either internal or external validation. 4 studies (23, 26, 32, 36) did not report the model calibration methods, while 10 studies (24, 25, 27–31, 33–35) provided calibration information, typically in the form of the Hosmer-Lemeshow test. See Tables 2–4.

**Table 2:** Model construction methods and performance.

Author (year)	Number of candidate variables	Variable selection method	Continuous variable handling	Missing data		Modeling approach	Model performance	
				Data	Handling methods		Performance	Calibration method
Godet et al. (2017) (23)	9	Univariate and multivariate stepwise regression	Retained in continuous form	1,276	Direct deletion	LR	A: 0.820	—
Sará-Ochoa et al. (2017) (24)	21	Univariate and multivariate analysis	Retained in continuous form	8	Mean imputation	—	A: 0.689	H–L test
Dos Reis et al. (2017) (25)	17	Univariate and multivariate analysis	Retained in continuous form	—	—	LR	A: 0.810	H–L test
Hsieh et al. (2018) (26)	37	Univariate and multivariate analysis	Retained in continuous form	—	—	ANN	A: 0.850	—
Bansal et al. 2022 (27)	21	Univariate and multivariate analysis	Retained in continuous form	—	—	LR	A: 0.720	H–L test
							B: 0.720	
Zhao et al. (2021) (28)	89	Recursive feature elimination	Retained in continuous form	—	Multiple imputation	ML	A1: 0.774	Calibration curve
							A2: 0.779	
							A3: 0.819	
							A4: 0.829	
							A5: 0.830	
							A6: 0.835	
							A7: 0.821	
							A8: 0.802	
							A9: 0.780	
							A10: 0.765	
							A11: 0.722	
							B1: 0.714	
							B2: 0.743	
							B3: 0.688	
							B4: 0.770	
							B5: 0.771	
							B6: 0.803	
							B7: 0.717	
							B8: 0.700	
							B9: 0.713	
							B10: 0.712	
							B11: 0.736	
Cinotti et al. (2022) (29)	20	Lasso regression	Retained in continuous form	—	Multiple imputation	LR	A: 0.790	H–L test, calibration curve
							B: 0.710	
Wang et al. (2023) (30)	29	Univariate and multivariate analysis	Retained in continuous form	—	—	LR	A: 0.926	H–L test
Li (2023) (31)	105	Univariate and multivariate analysis	Retained in continuous form	—	—	LR	A: 0.773	H–L test, calibration curve
							B: 0.738	
Yang et al. (2023) (32)	12	Univariate and multivariate analysis	Converted into a categorical variable	—	—	LR	A: 0.722	—
Zhao et al. (2023) (33)	22	Univariate and multivariate analysis	Retained in continuous form	—	—	LR	A: 0.870	H–L test, calibration curve
							B: 0.867	
Hu et al. (2024) (34)	18	Univariate and multivariate analysis	Partially converted to categorical variables	—	—	LR	A: 0.970	H-L test、Calibration curve
Xu et al. (2024) (35)	34	Recursive feature elimination	Retained in continuous form	—	—	LR, RF, SVM, XG Boost, Light GBM	A1: 0.766	Calibration curve
							A2:0.788	
							A3:0.805	
							A4:0.800	
							A5:0.799	
Sun et al. (2025) (36)	23	Univariate and multivariate analysis	Converted into a categorical variable	—	—	LR	A: 0.821	—

A, Model Development Group; B, Model Validation Group; LR, Logistic Regression; RF, Random Forest; ANN, Artificial Neural Network; ML, Machine Learning; DT, Decision; SVM, Support Vector Machine; XG Boost, eXtreme Gradient Boosting; Light GBM, ‌Light Gradient Boosting Machine. H–L test, Hosmer–Lemeshow.

A1: Logistic Regression; A2: Support Vector Machine; A3: Random Forest; A4: eXtreme Gradient Boosting; A5: ‌Light Gradient Boosting Machine; A6: Cat Boost; A7: Gradient Boosting Decision Tree; A8: AdaBoost; A9: Multi-Layer Perceptron; A10: K-Nearest Neighbor; A11: Naive Bayes; B1: Logistic Regression; B2: Support Vector Machine; B3: Random Forest; B4: eXtreme Gradient Boosting; B5: ‌Light Gradient Boosting Machine; B6: Cat Boost; B7: Gradient Boosting Decision Tree; B8: AdaBoost; B9: Multi-Layer Perceptron; B10: K-Nearest Neighbor; B11: Naive Bayes. “—”: No mentioned.

**Table 3:** Model validation and final predictors.

Author (year)	Validation method		Model presentation format	Final predictors
	Internal validation	External validation		
Godet et al. (2017) (23)	Bootstrap resampling	—	Risk score	Cough response, swallowing ability, swallowing reflex, CRS visual score
Sará-Ochoa et al. (2017) (24)	Bootstrap resampling	—	Model equation	BUN, oxygenation index, APACHE II, cumulative fluid balance, hemoglobin
Dos Reis et al. (2017) (25)	—	—	Risk score	Duration of mechanical ventilation, female, GCS motor score, secretions, cough response
Hsieh et al. (2018) (26)	Random split validation, K-fold cross-validation	—	—	TISS score, hemodialysis, rsbi, pre-extubation heart rate, pre-extubation oxygenation index, MEP
Bansal et al. 2022 (27)	Random split validation	—	Risk score	Duration of mechanical ventilation, body mass index, Glasgow Coma Scale score, mean airway pressure at 1 min of spontaneous breathing trial, fluid balance in the 24 h before extubation
Zhao et al. (2021) (28)	Random split validation	Spatial validation	Web calculator	Age, body mass index, stroke, heart rate, respiratory rate, mean arterial pressure, oxygen saturation, temperature, pH, central venous pressure, tidal volume, positive end-expiratory pressure, mean airway pressure, Pressure support level in pressure support ventilation mode, duration of mechanical ventilation, number of successful spontaneous breathing trials, fluid balance in the 24 h before extubation, type of antibiotics
Cinotti et al. (2022) (29)	Random split validation	—	Risk score	TBI, strong cough, gag reflex, swallowing ability, endotracheal suction ≤2 times per hour, GCS motor score, temperature on the day of extubation
Wang et al. (2023) (30)	—	Temporal validation	Model equation	Duration of mechanical ventilation, diaphragmatic excursion, diaphragmatic thickness variation, RSBI, inferior vena cava variability
Li (2023) (31)	Bootstrap resampling	Temporal validation	Nomogram	Duration of mechanical ventilation, APACHE II score, ROX index, COPD, PaO_2_, hemoglobin
Yang et al. (2023) (32)	—	—	Model equation	Duration of mechanical ventilation, age, GCS score, smoking index, MODS, underlying respiratory disease
Zhao et al. (2023) (33)	Random split validation	—	Nomogram	Duration of mechanical ventilation, APACHE II score, SOFA score, PaCO_2_, ventilator-induced diaphragmatic dysfunction
Hu et al. (2024) (34)	—	—	Nomogram	Duration of mechanical ventilation, age, COPD, smoking, D-dimer, oxygenation index
Xu et al. (2024) (35)	Five-fold cross-validation	—	Model equation	APACHE II, respiratory rate during SBT, GCS score, hemoglobin

**Table 4:** Classification table of predictors.

Predictive factor	Temporal attribute	Measurement method	Intervenability	Clinical significance
Duration of mechanical ventilation	Cumulative	Vital sign	No	Reflects the risk of respiratory muscle disuse atrophy
GCS score	Pre-extubation	Score	No	Related to the level of consciousness and airway protective capacity
APACHE II score	Pre-extubation	Score	No	Comprehensively assesses the severity of the disease
Age	Baseline	Demographic	No	Respiratory muscle reserve function declines with age
Hemoglobin	Pre-extubation	Lab	Yes	Oxygen delivery capacity affects respiratory muscle endurance
Respiratory rate	Pre-extubation	Vital Sign	Yes	Reflects the balance between respiratory drive and load
Serum albumin	Pre-extubation	Lab	Yes	Nutritional status is related to respiratory muscle protein synthesis

### 3.4 Model performance and included predictive factors

**Figure 2:**
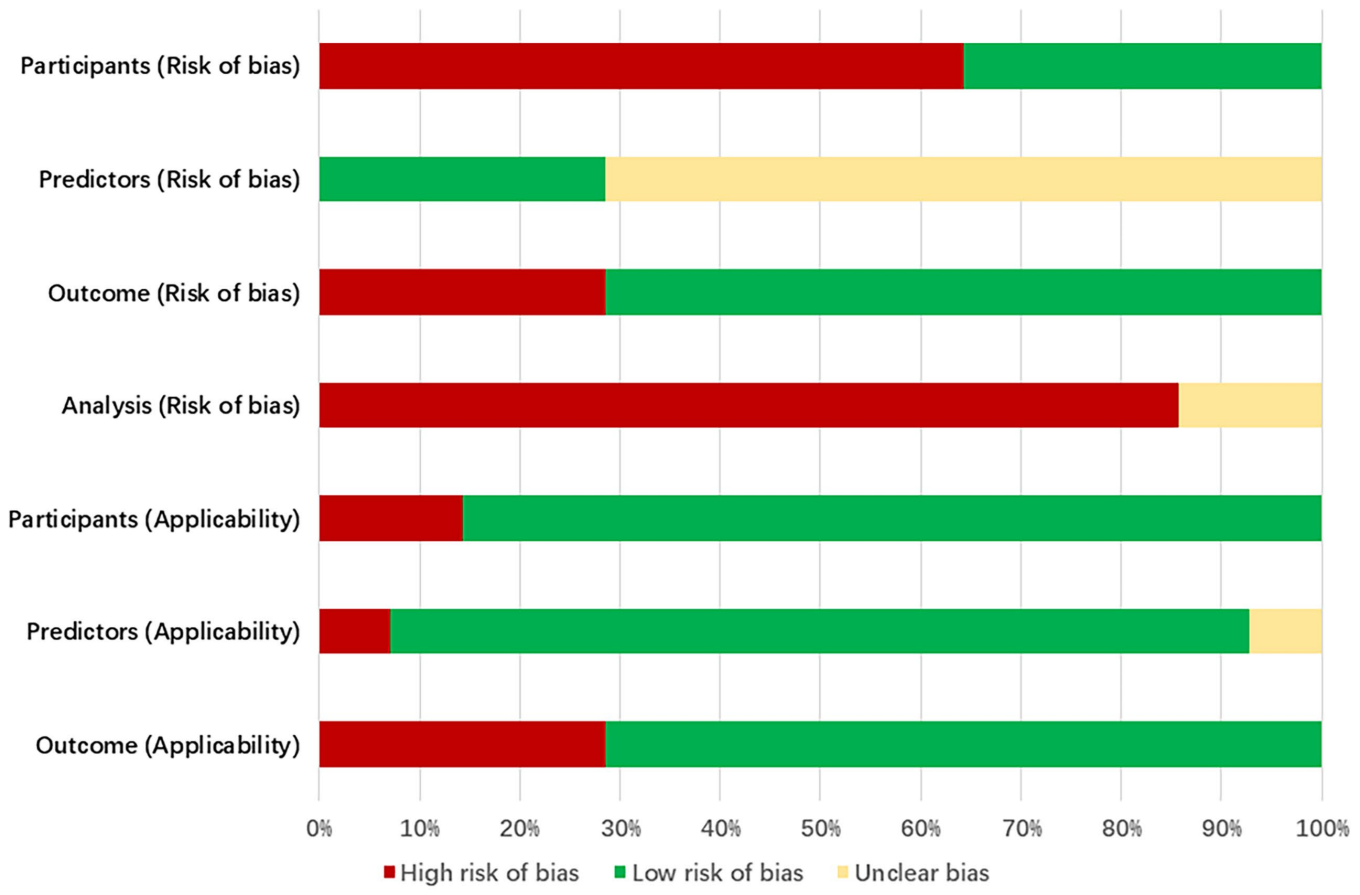
Results of the bias assessment of 14 studies.

Among the 14 studies included, the AUC values of the 28 models ranged from 0.688 to 0.970, with 26 models having an AUC greater than 0.7, indicating good predictive performance. Definitions of extubation failure and their time windows differed across studies; therefore AUCs are presented descriptively without quantitative synthesis, avoiding inflation of performance due to definitional heterogeneity. The final presentation formats of the models varied; 5 studies (24, 30, 32, 35, 36) presented the models in the form of equations, 4 studies (23, 25, 27, 29) utilized risk scores, 3 studies (31, 33, 34) presented the models as nomograms, and 1 study (26) did not specify the final presentation format of the model. The number of predictive factors included in the final models ranged from 4 to 17, with the top five most frequently occurring predictive factors being: mechanical ventilation duration, GCS score, APACHE II score, age, and hemoglobin. Predictive factors that appeared with a frequency of ≥2 times are shown in Figure 2, Tables 3–5

**Table 5:** Model external validation performance.

Author (year)	Data source	AUC	Calibration accuracy
Zhao et al. (2021) (28)	Cardiac Surgical ICU of Zhongshan Hospital, Fudan University	0.80 (0.74–0.83)	—
Wang et al. (2023) (30)	Department of Critical Care Medicine, Weifang People’s Hospital, Weifang, Shandong	0.924 (0.886–0.961)	H-L (*p* = 0.629)
Li (2023) (31)	Department of Critical Care Medicine, First Hospital of Lanzhou University, Lanzhou, Gansu	0.738 (0.630–0.846)	—
Sun et al. (2025) (36)	Department of Critical Care Medicine, Kaifeng Central Hospital, Kaifeng, Henan	—	—

H–L, Hosmer–Lemeshow test.

### 3.5 Assessment of bias risk and applicability

**Figure 3:**
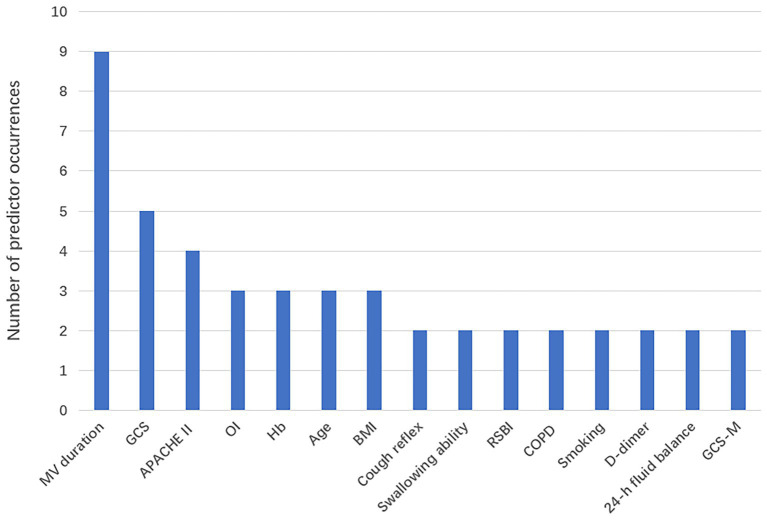
Predictor frequency distribution.

The bias assessment tool PROBAST was employed to evaluate the bias risk and applicability of the included literature. All studies were rated as having a high risk of bias, indicating methodological issues present in the development or validation process of the extubation failure models for ICU patients on mechanical ventilation. Specific results can be found in Figure 3, Table 6 and Appendix B.

**Table 6:** PROBAST results of the included studies.

Author (year)	ROB				Applicability			Overall	
	Participants	Predictors	Outcome	Analysis	Participants	Predictors	Outcome	ROB	Applicability
Godet et al. (2017) (23)	+	+	+	−	+	+	+	−	−
Sará-Ochoa et al. (2017) (24)	−	?	+	−	+	+	+	−	+
Dos Reis et al. (2017) (25)	+	?	+	?	+	+	+	−	−
Hsieh et al. (2018) (26)	+	+	−	−	+	+	−	−	+
Bansal et al. (2022) (27)	−	?	−	−	+	−	−	−	+
Zhao et al. (2021) (28)	+	+	+	−	+	+	+	−	+
Cinotti et al. (2022) (29)	+	+	−	−	+	+	−	−	+
Wang et al. (2023) (30)	−	?	+	−	+	+	+	−	+
Li (2023) (31)	−	?	+	?	+	+	+	−	+
Yang et al. (2023) (32)	−	?	+	−	+	?	+	−	+
Zhao et al. (2023) (33)	−	?	+	−	+	+	+	−	+
Hu et al. (2024) (34)	−	?	+	−	−	+	+	−	−
Xu et al. (2024) (35)	−	?	+	−	+	+	+	−	+
Sun et al. (2025) (36)	−	?	−	−	−	+	−	−	+

PROBAST, Prediction Model Risk of Bias Assessment Tool; ROB, risk of bias; + indicates low ROB/low concern regarding applicability; − indicates high ROB/high concern regarding application;? indicates unclear ROB/unclear concern regarding applicability.

#### 3.5.1 Bias in the field of study

9 studies (24, 27, 30–36) (64%, 9/14) exhibited a high risk of bias. This is attributed to the retrospective nature of the studies, which may introduce recall bias. Important predictive factors related to the failure of extubation from mechanical ventilation in ICU patients could not be obtained solely through the review of medical records.

#### 3.5.2 Bias in predictive factor

Research 5 studies (23, 25, 26, 28, 29) (36%, 5/14) were assessed as having a low risk of bias in the predictive factor domain, while 9 studies (24, 27, 30–36) (64%, 9/14) were rated as unclear. The reason for this is that the 5 studies were prospective in nature, with the measurement of predictive factors conducted prior to the occurrence of outcomes, utilizing a blind method by default. The remaining 9 studies were retrospective, and it remains unclear whether the assessment of predictive factors was conducted without knowledge of the outcome data.

#### 3.5.3 Bias in outcome domains

11 studies (23–25, 28, 30–36) (79%, 11/14) were rated as low risk in the predictor domain, while 3 studies (26, 27, 29) (21%, 3/14) were rated as high risk. This may be because ten studies not only utilized standardized guidelines but also had clear and consistent definitions of outcome indicators. The remaining three studies were rated high risk due to offline assessment of outcomes exceeding 48 h.

#### 3.5.4 Analysis of bias in the field

Fourteen studies exhibited a high risk of bias in the analysis. The issues identified include: ① In 13 studies (23–27, 29–36), the number of outcome events was insufficient (EPV < 20); ② 3 studies (32, 34, 36) improperly transformed continuous variables into categorical variables, indicating an inappropriate variable handling method; ③ 10 studies (25–27, 30–36) did not report missing data and the methods for handling it; ④ 11 studies (23–27, 30–34, 36) selected predictive factors based on univariate analysis without employing appropriate variable selection methods; ⑤ 3 studies (25, 32, 34) did not perform internal or external validation of the models; ⑥ None of the 14 studies addressed the issue of model overfitting or underfitting.

#### 3.5.5 Applicability assessment

11 studies (24, 26–33, 35, 36) demonstrated overall good applicability, while 3 studies (23, 25, 34) exhibited relatively low overall applicability. Among them, 2 studies (23, 25) were limited to mechanically ventilated patients with traumatic brain injuries in the ICU, and one study (34) focused on elderly patients with severe pneumonia.

## 4 Discussion

### 4.1 Quality of research on extubation failure risk prediction models is acceptable but contains certain biases

This study provides a comprehensive analysis of prediction models to identify the extubation failure risk in adult patients undergoing mechanical ventilation in the ICU. A total of 28 prediction models were included, with AUC values ranging from 0.688 to 0.970. Among these, 26 models exhibited an AUC greater than 0.7, indicating good predictive performance. The high risk of bias is primarily concentrated in the analytical domain, mainly due to insufficient outcome event numbers, improper handling of variables, the selection of predictive factors based on univariate analysis, failure to report missing data information, incomplete model performance evaluation, and lack of reporting on model fit.

#### 4.1.1 Data sources

In terms of research type, this study includes 9 retrospective cohort studies (24, 27, 30–36). The predictive factors incorporated into the model may not be comprehensive, and there is a potential risk of data omission, which could lead to biased results. In prospective studies, the measurement of predictive factors occurs before the outcomes, effectively standardizing the assessment methods for these factors. This standardization significantly enhances the reliability of the model results. The PROBAST evaluation tool suggests that to mitigate the risk of overfitting in model development research, the number of outcome events should be at least 20 times the number of candidate predictors. This implies that the events per variable (EPV) should exceed 20. Given that the risk prediction model for extubation failure in ICU patients on mechanical ventilation includes numerous candidate predictors, it becomes challenging to satisfy the EPV > 20 criterion. Consequently, future model studies should include a sufficiently large sample size. Future research should prioritize the pre-selection of clinically significant and potentially predictive variables through methods such as clinical expertise, literature review, or univariate analysis before formal modeling. It is generally advised that the final model include no more than 10 to 15 predictor variables, ensuring that the events per variable (EPV) ratio approaches or exceeds 20. This approach serves to mitigate the risk of overfitting.

#### 4.1.2 Data analysis

① In the handling of continuous variables, Collins et al. (37) point out that when constructing risk prediction models, converting continuous variables into two or more categorical variables can increase the risk of the model. Among the studies included in this research, 3 studies (32, 34, 36) converted continuous variables into categorical variables, which may lead to a higher risk of bias in the included studies. Future studies should retain continuous predictors in their original form or model them with flexible functions such as restricted cubic splines; this preserves information, avoids arbitrary cut-point bias, and improves both discrimination and calibration. Owing to substantial heterogeneity in data sources, candidate predictor sets, and modelling methods across studies, we could not directly compare AUCs or calibration between models that kept variables continuous and those that converted them to categories. Future work should use a single dataset and an identical modelling pipeline to test both approaches and thereby quantify their true effects on discrimination and calibration. ② In the realm of missing data handling, 12 studies (25–36) did not report any missing data, whereas 1 study (23) explicitly excluded subjects with missing data, which indicates inadequate handling of this issue. Such an approach may result in the loss of valuable hidden information within the excluded subjects, potentially leading to bias in the model. Missing data can significantly impact the quality of data analysis and the accuracy of models, making the preprocessing of missing data particularly important. The PROBAST guidelines suggest that missing values should not be deleted directly; instead, multiple imputation should be employed (38). Multiple imputation methods can effectively reduce the adverse effects of missing data on statistical analysis and model stability, thereby improving research accuracy and reliability (39). Future researchers should provide a comprehensive account of missing values and the methods employed to handle them during the model construction process. It is recommended that multiple imputation techniques be utilized to address these missing values effectively. ③ Selection of Predictors in this study, the predictors were primarily identified through univariate analysis to find statistically significant variables, followed by Logistic Regression analysis to incorporate these significant variables into the model. This method of screening predictors can reduce the workload but may overlook important risk factors. Therefore, it is recommended that future research utilize stepwise regression to mitigate multicollinearity issues effectively. LASSO regression, which employs the least absolute shrinkage and selection operator, can perform parameter estimation and variable selection simultaneously. ④ In terms of model validation, only 2 studies (28, 31) conducted internal and external validation, while 7 studies (23, 24, 26, 27, 29, 33, 35) performed only internal validation (Bootstrap resampling and random grouping validation) 0.2 studies (30, 36) conducted only external validation, and three studies (25, 32, 34) did not perform either internal or external validation. Therefore, future researchers may choose high-quality predictive models for external validation for the risk of extubation failure in ICU patients on mechanical ventilation based on the results of this study.

Despite the high risk of bias associated with all studies, which somewhat limits the clinical application of the models, valuable insights can still be gained from the recommendation processes of the models. Cinotti et al. (29) conducted a prospective multicenter study involving 1,512 neurocritical patients across 73 intensive care units (ICUs) in 18 countries, effectively mirroring clinical decision-making scenarios. The authors utilized LASSO regression for the data-driven automatic selection of candidate variables and employed ten-fold cross-validation to identify independent predictive factors. This approach effectively addresses multicollinearity issues while maintaining the model’s simplicity and robustness, establishing a strong foundation for subsequent extrapolation applications in diverse medical environments. Zhao et al. (28) conducted a study utilizing the MIMIC-IV database, which comprised a training set of 16,189 patients, and performed an independent prospective validation with 502 patients from the cardiac surgery ICU at Zhongshan Hospital, affiliated with Fudan University. This methodology effectively balanced sample size and generalizability, mitigating the overfitting risk. The CatBoost algorithm inherently accommodates missing values and categorical variables, eliminating the necessity for additional imputation and dummy variable encoding. The study ultimately retained only 17 readily obtainable bedside indicators by integrating a SHAP-based recursive feature elimination strategy. The internal validation set achieved an area under the receiver operating characteristic curve (AUROC) of 0.835. In contrast, the external validation set reached an AUROC of 0.803, significantly surpassing traditional scoring systems such as the RSBI and SOFA (*p* < 0.01). Furthermore, the research team developed a plug-and-play web-based prediction tool that outputs risk probabilities in real-time based on input variables, thereby providing a visual and generalizable digital foundation for clinical extubation decisions.

### 4.2 Predictive factors for extubation failure

Variations and commonalities arise due to differences in research types and the variables included, leading to inconsistencies in the predictive factors identified across various studies. Nonetheless, this study identifies commonalities among the predictive factors recognized in different research efforts. Specifically, this research explores five risk predictive factors that frequently appear: duration of mechanical ventilation, Glasgow Coma Scale (GCS) score, APACHE II score, age, and hemoglobin levels. Prolonged mechanical ventilation can result in diaphragmatic disuse atrophy and decreased contractile function. Research indicates that diaphragmatic dysfunction occurs in up to 37% of long-term mechanical ventilation patients and is significantly associated with weaning failure (40, 41). Prolonged mechanical ventilation can lead to ICU-acquired muscle weakness, which decreases the contractile strength of respiratory muscles, including the diaphragm and accessory respiratory muscles. This muscle weakness significantly increases the risk of weaning failure by a factor ranging from 2.64 to 19.07 (42). Prolonged mechanical ventilation increases the risk of complications, including ventilator-associated pneumonia (VAP) and barotrauma, which may further exacerbate respiratory function impairment (43).

The APACHE II scoring system is used to assess the severity of illness and mortality risk in ICU patients. A higher score indicates a more severe condition and an increased risk of death. A higher APACHE II score indicates more severe systemic physiological disturbances and organ failure, which can directly increase the risk of offline failure through multiple pathways. Patients with elevated scores frequently experience severe hypoxemia, acidosis, hemodynamic instability, and multiple organ dysfunction, resulting in an imbalance between respiratory work and oxygen consumption (44). The severe inflammatory response, malnutrition, and accelerated muscle protein breakdown lead to a synchronous decline in the strength of the diaphragm and peripheral muscles (45). A high APACHE II score frequently suggests the necessity for deep sedation, larger doses of vasopressors, or continuous renal replacement therapy (46). These interventions can suppress the respiratory drive of the central nervous system, thereby delaying the recovery of consciousness and re-establishing airway protective reflexes. Consequently, this may lead to a significant reduction in the success rate of spontaneous breathing trials and an increased likelihood of re-intubation and mortality in the ICU (47).

Vidotto et al. (48) found that when the patient’s GCS is less than 8, the extubation success rate drops sharply to 33%, indicating that the level of consciousness is a key threshold variable in predicting weaning outcomes. The degree of consciousness impairment is linearly negatively correlated with the integrity of airway protection and respiratory drive. As the GCS score decreases, the cough reflex arc is inhibited, and the sensitivity of the respiratory center to hypercapnia and hypoxemia significantly diminishes. Consequently, patients struggle to maintain adequate tidal volume and rhythmic stability during spontaneous breathing trials, which leads to an extension of mechanical ventilation duration and an exponential increase in the risk of extubation failure (49). A decrease in the GCS score may also be accompanied by dysfunction in other organ systems, such as an increase in the SOFA score, further complicating the weaning process (50). Therefore, healthcare professionals should enhance airway management for patients with altered consciousness based on the GCS score and proactively implement targeted interventions to reduce weaning difficulties.

As individuals age, their bodily functions gradually decline. With increasing age, a person’s respiratory reserve diminishes, leading to a heightened risk of decompensation. As age increases, there is a reduction in type II muscle fibers in the diaphragm, accompanied by mitochondrial dysfunction. This results in an exponential decline in the endurance and strength of the respiratory muscles, leading to a higher likelihood of diaphragm fatigue during spontaneous breathing tests. Consequently, this triggers instability in central-ventilatory coupling (51). Immunosenescence and chronic low-grade inflammation significantly increase the susceptibility of elderly patients to volume overload, ventilator-associated diaphragm dysfunction, and nosocomial infections, triggering a systemic inflammatory response syndrome and delaying diaphragm repair (52). Elderly patients frequently demonstrate a decline in cardiac functional reserve, pulmonary vascular remodeling due to arteriosclerosis, and neurodegenerative disorders. These comorbidities further exacerbate the adverse effects of aging on the extubation outcomes of ICU patients receiving mechanical ventilation by diminishing cardiopulmonary coupling efficiency, extubation respiratory drive, and impairing central integration capabilities (53).

Hemoglobin, a crucial carrier of oxygen in the bloodstream, exhibits abnormal levels that significantly impact the extubation outcomes of patients undergoing mechanical ventilation in the ICU. In a state of low hemoglobin, the oxygen supply capability of tissues in patients decreases, leading to impaired respiratory muscle function, affecting respiratory drive and endurance (54). Relevant research indicates that a decrease in hemoglobin concentration directly reduces arterial blood oxygen content, leading to a hypoxic state in peripheral tissues and respiratory muscles. This hypoxia not only diminishes the contractile ability of the respiratory muscles but may also extend the duration of mechanical ventilation required for patients (55). Conversely, elevated hemoglobin levels adversely affect pulmonary blood circulation by increasing blood viscosity. This increase in blood viscosity results in heightened microcirculatory resistance, consequently diminishing the adequate perfusion of lung tissue and further impairing gas exchange efficiency (56). Additionally, elevated hemoglobin levels may promote the formation of microthrombi, thereby exacerbating pulmonary vascular resistance and potentially leading to offline failure.

## 5 Limitations

Although this study provides a comprehensive summary of the prediction models for extubation failure in ICU patients on mechanical ventilation, certain limitations persist. Firstly, this study exclusively included retrievable literature in both Chinese and English, which may introduce publication bias. Secondly, the imposed English/Chinese language restriction could have omitted relevant studies published in other languages, potentially limiting the generalisability of our findings. Thirdly, due to variations in inclusion criteria and study designs across different studies, only a qualitative analysis of the research results was conducted, precluding a quantitative analysis. This review could not standardise the original definitions; a consensus core outcome (e.g., re-intubation within 48 h) with broader criteria analysed separately in sensitivity analyses should be adopted in future work.

## 6 Conclusion

This paper comprehensively evaluates predictive models for extubation failure in patients undergoing invasive mechanical ventilation in the ICU. The findings suggest that current models are susceptible to bias due to several methodological flaws identified during the model development process, and some models lack external validation. To enhance the quality of future research, the research team should adhere to the PROBAST and TRIPOD guidelines for model construction, design, and reporting processes. Additionally, validating existing models across various regions will improve the external generalizability of risk prediction models. The research community should prioritise independent replication of the remaining models rather than creating new ones, so as to consolidate the evidence base before widespread implementation.

## Data Availability

The original contributions presented in the study are included in the article/Supplementary material, further inquiries can be directed to the corresponding author.
