# Supplementary material for: Risk prediction models for extubation failure in critically ill patients on mechanical ventilation: a systematic review
Source: Front Med (Lausanne). 2025 Nov 20;12:1695394. doi: 10.3389/fmed.2025.1695394 (PMC12675440; doi:10.3389/fmed.2025.1695394)
Supplement: Supplementary file 1 [file Data_Sheet_1.docx]

| Study | Participants | | Predictors | | | Outcome | | | | | | Analysis | | | | | | | | | Overall Evaluation |
| --- | --- | --- | --- | --- | --- | --- | --- | --- | --- | --- | --- | --- | --- | --- | --- | --- | --- | --- | --- | --- | --- |
|  | ① | ② | ③ | ④ | ⑤ | ⑥ | ⑦ | ⑧ | ⑨ | ⑩ | ⑪ | ⑫ | ⑬ | ⑭ | ⑮ | ⑯ | ⑰ | ⑱ | ⑲ | ⑳ |  |
| Godet 2017 | Y | Y | Y | Y | PY | Y | Y | Y | Y | Y | Y | Y | Y | PY | Y | Y | N | Y | Y | Y | — |
| Sará-Ochoa 2017 | N | Y | PY | PY | Y | Y | Y | Y | Y | NI | Y | Y | PY | Y | Y | N | N | Y | PY | Y | — |
| Dos Reis 2017 | Y | Y | Y | Y | Y | Y | Y | Y | Y | NI | Y | Y | PY | Y | Y | PY | N | Y | PY | Y | — |
| Hsieh 2018 | Y | Y | Y | Y | Y | Y | Y | Y | Y | N | Y | Y | PY | Y | Y | PY | N | Y | PY | Y | — |
| Bansal 2021 | N | Y | Y | NI | Y | Y | Y | Y | Y | N | Y | Y | Y | PY | Y | PY | N | Y | PY | Y | — |
| Zhao 2021 | Y | Y | Y | Y | Y | Y | Y | Y | Y | Y | Y | Y | Y | Y | Y | Y | N | Y | PY | Y | — |
| Cinotti 2022 | Y | Y | Y | Y | Y | Y | Y | Y | Y | N | Y | Y | Y | PY | Y | N | PY | Y | Y | Y | — |
| Wang 2023 | N | Y | Y | PY | Y | Y | Y | Y | Y | Y | Y | Y | Y | Y | Y | N | PY | Y | Y | Y | — |
| Li 2023 | N | Y | Y | PY | Y | Y | Y | Y | Y | Y | Y | Y | Y | PY | Y | N | PY | Y | PY | Y | — |
| Yang 2023 | N | Y | Y | PY | Y | Y | Y | Y | Y | Y | Y | Y | Y | Y | Y | N | PY | Y | Y | Y | — |
| Zhao 2023 | N | Y | Y | NI | Y | Y | Y | Y | Y | Y | Y | Y | Y | Y | Y | N | PY | Y | PY | Y | — |
| Hu 2024 | N | Y | Y | PY | Y | Y | Y | Y | Y | Y | Y | Y | Y | N | Y | N | Y | Y | Y | Y | — |
| Xu 2024 | N | Y | Y | PY | Y | Y | Y | Y | Y | Y | Y | Y | Y | Y | Y | N | N | Y | Y | Y | — |
| Sun 2025 | N | Y | Y | PY | Y | Y | Y | Y | Y | N | Y | Y | Y | Y | Y | N | Y | Y | Y | Y | — |

**Appendix Table 1**

**Note:** Y: Yes; PY: Possibly yes; N: No; PN: Possibly no; NI: Insufficient information; −: High risk of bias. ① Was an appropriate data source used, e.g. cohort, randomised controlled trial or nested case–control data? ② Were appropriate inclusion and exclusion criteria applied to all study participants? ③ Were predictors for all participants defined and assessed in a similar way? ④ Were predictors assessed without knowledge of the outcome data? ⑤ Were all predictors available when the model was used?⑥ Was the outcome appropriately determined? ⑦ Was a pre-specified or standard definition of the outcome used? ⑧ Were predictors excluded from the outcome definition? ⑨ Were outcomes defined and determined in a similar manner? ⑩ Was the outcome determined without knowledge of predictor information?

⑪ Was the time interval between predictor assessment and outcome determination appropriate? ⑫ Was the number of outcome events adequate? ⑬ Were continuous and categorical variables handled appropriately? ⑭ Were all enrolled participants included in the analysis? ⑮ Were participants with missing data handled appropriately? ⑯ Was predictor selection based on univariable analysis avoided? ⑰ Was data complexity (e.g. censoring, competing risks, control sampling) accounted for? ⑱ Were model performance measures assessed appropriately? ⑲ Were issues of model over-fitting or under-fitting considered? ⑳ Are the predictors in the final model and their assigned weights related to the results from the multivariable analysis?
