# Supplementary material for: Risk prediction models for extubation failure in critically ill patients on mechanical ventilation: a systematic review
Source: Front Med (Lausanne). 2025 Nov 20;12:1695394. doi: 10.3389/fmed.2025.1695394 (PMC12675440; doi:10.3389/fmed.2025.1695394)
Supplement: Supplementary file 2 [file Table_1.DOCX]

Search date: 2025.8.8

**Table S1.** Search terms in PubMed

| Databases | Step | Searches | Results |
| --- | --- | --- | --- |
| PubMed | #1 | ("Intensive Care Units"[Mesh]) OR ((((((intensive care unit[Title/Abstract]) OR (intensive care[Title/Abstract])) OR (critical care[Title/Abstract])) OR (critical illness[Title/Abstract])) OR (critically ill[Title/Abstract])) OR (unit intensive care[Title/Abstract])) | 334,383 |
|  | #2 | (("Intubation, Intratracheal"[Mesh])OR ("Respiration, Artificial"[Mesh]))OR((((((((respiration, artificial[Title/Abstract]) OR (artificial respiration*[Title/Abstract]))OR (mechanical ventilation*[Title/Abstract])) OR (intratracheal intubation*[Title/Abstract]))OR intratrachealTitle/Abstract])) OR (intubation,OR(intubations,intratracheal[Title/Abstract])) OR (intubation,endotrachealTitle/Abstract)) OR (intubations, endotracheal[Title/Abstract\|) OR (endotracheal intubation*[Title/Abstract])) OR (tracheal intubation[Title/Abstract])) | 188,434 |
|  | #3 | (((((((Risk Assessment[Title/Abstract])) OR (predict* model[Title/Abstract])) OR (prognos* model[Title/Abstract])) OR (risk predict*[Title/Abstract])) OR (risk score[Title/Abstract])) OR (predictive factor*[Title/Abstract])) OR (risk factor*[Title/Abstract]) | 1,122,307 |
|  | #4 | #1 AND #2 AND #3 | 6509 |
